# Percutaneous Management of Desmoid Tumors

**DOI:** 10.1007/s11912-026-01821-3

**Published:** 2026-08-22

**Authors:** Gina P. Landinez, Shakthi Kumaran Ramasamy, Xiao Wu, Kiyon Naser-Tavakolian, Abin Sajan, Areeb Siddiqui, John Moody, Kirema Garcia-Reyes, Junaid Raja, Venkatesh P. Krishnasamy

**Affiliations:** 1 Department of Cardiovascular Sciences, Herbert Wertheim College of Medicine, Florida International University, Miami, FL USA; 2 Baptist Health Heart and Vascular, Miami Cardiac and Vascular Institute, Miami, FL USA; 3 Department of Radiology and Biomedical Imaging, University of California San Francisco, San Francisco, CA USA; 4 Department of Vascular and Interventional Radiology, Columbia University, New York, USA; 5 The Johns Hopkins School of Medicine, Baltimore, MD USA; 6 Heersink School of Medicine, University of Alabama at Birmingham, Birmingham, AL USA; 7 Department of Diagnostic, Molecular & Interventional Radiology, Icahn School of Medicine at Mount Sinai, New York, NY USA; 8 Division of Vascular and Interventional Radiology, University of Alabama Birmingham, Birmingham, AL USA

**Keywords:** Desmoid tumor, Aggressive fibromatosis, Cryoablation, Radiofrequency ablation, Microwave ablation, Irreversible electroporation, High-intensity focused ultrasound, Transcatheter embolization, Nirogacestat, Interventional radiology, NCCN

## Abstract

**Purpose of Review:**

To provide a comprehensive review of percutaneous image-guided techniques in the management of desmoid tumors including evolving thermal and non-thermal ablation options, as well as trans-arterial catheter options. Beyond each treatment modality, we aim to highlight considerations related to patient selection, technical components, and the growing interplay between percutaneous therapies and emerging systemic therapies.

**Recent Findings:**

Percutaneous ablation, particularly cryoablation, has emerged as a safe and effective treatment for extra-abdominal desmoid tumors, with prospective data demonstrating durable disease control and significant improvements in pain and functional status. A meta-analysis of 694 desmoid tumors treated with thermal ablation reported pooled local control of 88%, symptom relief in 90% of patients, and major complication rates below 5%. Transarterial doxorubicin-eluting embolization has shown promise in both adult and pediatric settings, while bland particle and radioembolic approaches remain under investigation. Irreversible electroporation is emerging as a non-thermal alternative near critical structures. The National Comprehensive Cancer Network (NCCN) Soft Tissue Sarcoma Guidelines now formally incorporate cryoablation along with embolization into the treatment algorithm for progressive, morbid, or symptomatic extra-abdominal desmoid tumors, marking a significant milestone in the recognition of percutaneous techniques as standard-of-care options.

**Summary:**

Percutaneous image-guided techniques now offer local tumor control with rates similar to surgical resection and a favorable safety profile. Some of these percutaneous techniques are endorsed alongside systemic therapy and radiation as primary options for progressive or symptomatic extra-abdominal desmoid tumors per various guidelines. An important consideration involves the level of data, comprised predominantly of retrospective data, without direct comparative date. The ongoing prospective CRYODESMO-02 trial will help define optimal sequencing and strengthen the evidence base underpinning guideline recommendations.

## Introduction

Desmoid tumors are rare clonal neoplasms characterized by local invasiveness without metastatic potential, with an annual incidence of approximately 3–4 cases per million in the U.S. population [1]. They typically affect younger patients, show a female predominance, and can arise at virtually any anatomic site [2]. The clinical course is highly variable, ranging from spontaneous regression to persistent local progression causing significant pain, functional impairment, and diminished quality of life [3, 4].

Management has undergone fundamental transformation over the past two decades. Practice initially mirrored that used for soft tissue sarcomas, but recognition that margin positivity exceeds 40%, that 5-year recurrence-free survival after resection is only approximately 53%, and that margin status does not consistently predict recurrence [5, 6], together with observations that 20–30% of tumors spontaneously regress [7, 8], prompted a shift toward watchful waiting. A multicenter EMSOS study of 388 patients confirmed comparable outcomes between surgery (36.6% recurrence) and active surveillance (40.2% progression) and identified pain as a novel predictor of progression during observation [9]. Active surveillance is now the preferred initial strategy for minimally symptomatic or non-critically located disease.

When active treatment is warranted, the therapeutic landscape now includes systemic agents (tyrosine kinase inhibitors, gamma-secretase inhibitors), radiation, surgery, and a rapidly expanding portfolio of percutaneous image-guided techniques [10–12]. A critical milestone in this evolution was the incorporation of ablation and embolization into the NCCN Soft Tissue Sarcoma Guidelines as recognized local therapy options for desmoid tumors [13]. Both percutaneous treatment options listed alongside systemic therapy and definitive radiation as options for progressive, morbid, or symptomatic extra-abdominal desmoid tumors - abdominal wall, pelvis, trunk/extremity, head/neck, and intrathoracic locations - with surgery positioned as the less-preferred alternative [13]. DEB-TACE is specifically recognized as a reasonable alternative for selected extra-abdominal desmoid tumors when cryoablation is not technically feasible. The Desmoid Tumor Working Group (DTWG) 2024 consensus similarly recognizes local ablative techniques as valid treatment options [14].

This review provides a comprehensive update on percutaneous management of desmoid tumors including cryoablation, radiofrequency ablation, microwave ablation, irreversible electroporation, chemical ablation, high-intensity focused ultrasound, and transarterial embolization, with emphasis on efficacy, safety, patient selection, and the integration of these techniques with systemic therapy.

### Overview of Percutaneous Ablation Modalities

Image-guided ablation achieves tissue destruction through the delivery of extreme temperatures, electrical fields, acoustic energy, or cytotoxic agents. Ultrasound, CT, and MRI provide the basis for procedural planning, probe placement, real-time monitoring, and post-treatment assessment, enabling targeted tumor destruction while sparing adjacent critical structures [15]. The expanding role of interventional radiology in desmoid tumor care, and the full armamentarium of minimally invasive options, has been reviewed recently [16].

Each modality occupies a distinct niche. Chemical ablation, the earliest described percutaneous technique for desmoid tumors, uses direct intratumoral injection of cytotoxic agents such as acetic acid, inducing coagulative necrosis through protein denaturation and collagen dissolution [10]. Clark reported CT-guided intratumoral injection of 50% acetic acid in two patients with refractory disease, achieving complete and partial responses with durable control at 24–36 months. Though largely superseded by thermal modalities with more predictable ablation zones, this work established the framework for percutaneous desmoid management [10]. Cryoablation, uses the expansion of pressurized argon to generate lethal cold temperatures via the Joule-Thomson effect. Radiofrequency ablation (RFA) induces coagulative necrosis through frictional heating from an alternating electric current delivered via a percutaneous electrode [17]. Microwave ablation (MWA) uses electromagnetic energy to generate heating, offering shorter procedure times and reduced susceptibility to heat-sink effects compared with RFA [18]. Irreversible electroporation (IRE), a non-thermal modality, uses brief high-voltage electrical pulses to induce cell membrane permeabilization and apoptosis while better preserving extracellular matrix and neurovascular structures. High-intensity focused ultrasound (HIFU) is non-invasive, using focused acoustic energy to heat tissues to ablative temperatures under MRI or ultrasound guidance [19]. Transarterial embolization uses the typical hypervascularity of desmoid tumors, delivering cytotoxic drugs, bland particles, or radionuclides to induce ischemia, necrosis, and, where applicable, local drug exposure [20].

### Radiofrequency Ablation

RFA was among the earliest thermal modalities applied to desmoid tumors, with case reports demonstrating feasibility for both abdominal wall and intra-abdominal tumors in familial adenomatous polyposis with multiple sessions [17, 21, 22]. RFA has technical limitations in desmoid tumors: reduced efficacy against dense collagenous stroma due to high tissue impedance, greater risk of thermal injury compared with cryoablation, and susceptibility to heat-sink effects. Cryoablation has largely superseded RFA for desmoid treatment, although RFA remains viable where cryoablation is unavailable.

### Microwave Ablation

Desmoid-specific MWA data remain relatively sparse. A retrospective study of 9 patients (mean diameter 10.4 cm) treated with MWA reported 70.4% mean volume reduction, complete response in two patients, and functional improvement in eight of nine [18]. Zhang et al. recently reported the first pediatric experience, treating 8 children (mean age 9.2 years) with 10 recurrent desmoid tumors across 11 ultrasound-guided MWA sessions; mean volume reduction was 55.86% with an 80% overall effectiveness rate, significant pain reduction, and only one grade 1 peroneal nerve injury that recovered [23].

MWA offers hypothetical advantages of higher intratumoral temperatures and reduced heat-sink susceptibility compared with RFA, with shorter procedure times. However, the overall desmoid-specific experience remains limited, and comparative studies with cryoablation are needed before its role can be fully defined.

### Cryoablation

#### Physiology and Technical Considerations

While the classic “lethal ice” threshold is approximately − 20 °C, modern cryoprobes generate considerably colder central temperatures, typically ranging from − 140 °C to − 190 °C at the probe tip. This distinction is clinically important as the visible ice ball margin on CT or MRI corresponds to the 0 °C isotherm, whereas the lethal − 20 °C isotherm lies several millimeters inside the visible edge. Targeting the ice ball boundary to the tumor margin therefore undertreats the periphery; adequate oncologic coverage requires extending the visible ice ball 5–10 mm beyond the tumor border to ensure the lethal isotherm encompasses the entire lesion. This principle is reinforced by the observation across multiple series that recurrences after cryoablation occur predominantly at the tumor periphery [24, 25].

Cryoablation has several intrinsic advantages for desmoid tumors. The ice ball is clearly visible on CT and MRI, and its superficial and lateral margins can also be visualized on ultrasound, enabling real-time multimodal monitoring [11, 12]. Multiple cryoprobes can be organized to sculpt the ice ball to complex tumor geometries, and staged sessions allow management of particularly large lesions.

### Cryoablation

#### Prospective Evidence

The strongest prospective evidence comes from CRYODESMO-01, a multicenter phase II trial of 50 patients with non-abdominopelvic desmoid tumors (median diameter 10.0 cm) progressing after medical treatment [24]. At 12 months, complete response was achieved in 28.6%, partial response in 26.2%, and stable disease in 31%, with sustained improvements in pain and functional status. At a median follow-up of 31 months, median progression-free survival was not reached; largest tumor diameter was the only predictor of treatment failure, and all recurrences occurred at the tumor periphery [24, 25]. These results, together with the growing retrospective experience, contributed to the inclusion of cryoablation in the DTWG 2024 guidelines and, subsequently, in the NCCN algorithm as a recognized option for progressive extra-abdominal disease [13]. The ongoing CRYODESMO-02 trial (NCT06081400) will provide the first randomized comparison of cryoablation versus standard medical therapy as first-line treatment following watchful waiting and will be pivotal in elevating the level of evidence supporting percutaneous ablation.

### Cryoablation

#### Retrospective Evidence

Multiple retrospective series spanning up to 10 years consistently demonstrate disease control rates of 80–92% by mRECIST, symptomatic relief in 89–97%, and 1- and 3-year progression-free survival of approximately 85% and 77–83% respectively [26–32]. A propensity-matched comparison demonstrated comparable two-year local recurrence-free survival between cryoablation and surgery (59% vs. 71%) [33].

Complete ice ball coverage consistently predicts superior outcomes; the largest single-center experience (75 patients) confirmed that complete coverage prolonged progression-free survival (OR 7.14; *p* < 0.05) and identified prior tyrosine kinase inhibitor therapy as the only predictor of severe complications (OR 53.8; *p* < 0.05), an important sequencing consideration [34]. Johnston et al. reported no recurrence when complete coverage was achieved, with all progression occurring outside ablation zones [32]. Bouhamama et al. demonstrated that analgesic benefit was independent of curative versus debulking intent, although complete coverage yielded significantly lower recurrence rates [35]. In the pediatric population, Shaikh et al. reported 100% pain relief and 90% objective response in 21 patients with only minor complications [36]. Two systematic reviews report pooled non-progressive disease rates exceeding 85%, 1- and 3-year progression-free survival of approximately 85% and 78%, objective response rates of 80%, mean pain reduction of 79%, and major complication rates below 5% [37, 38].

### Cryoablation

#### Expanding Indications

**Fig. 1:**
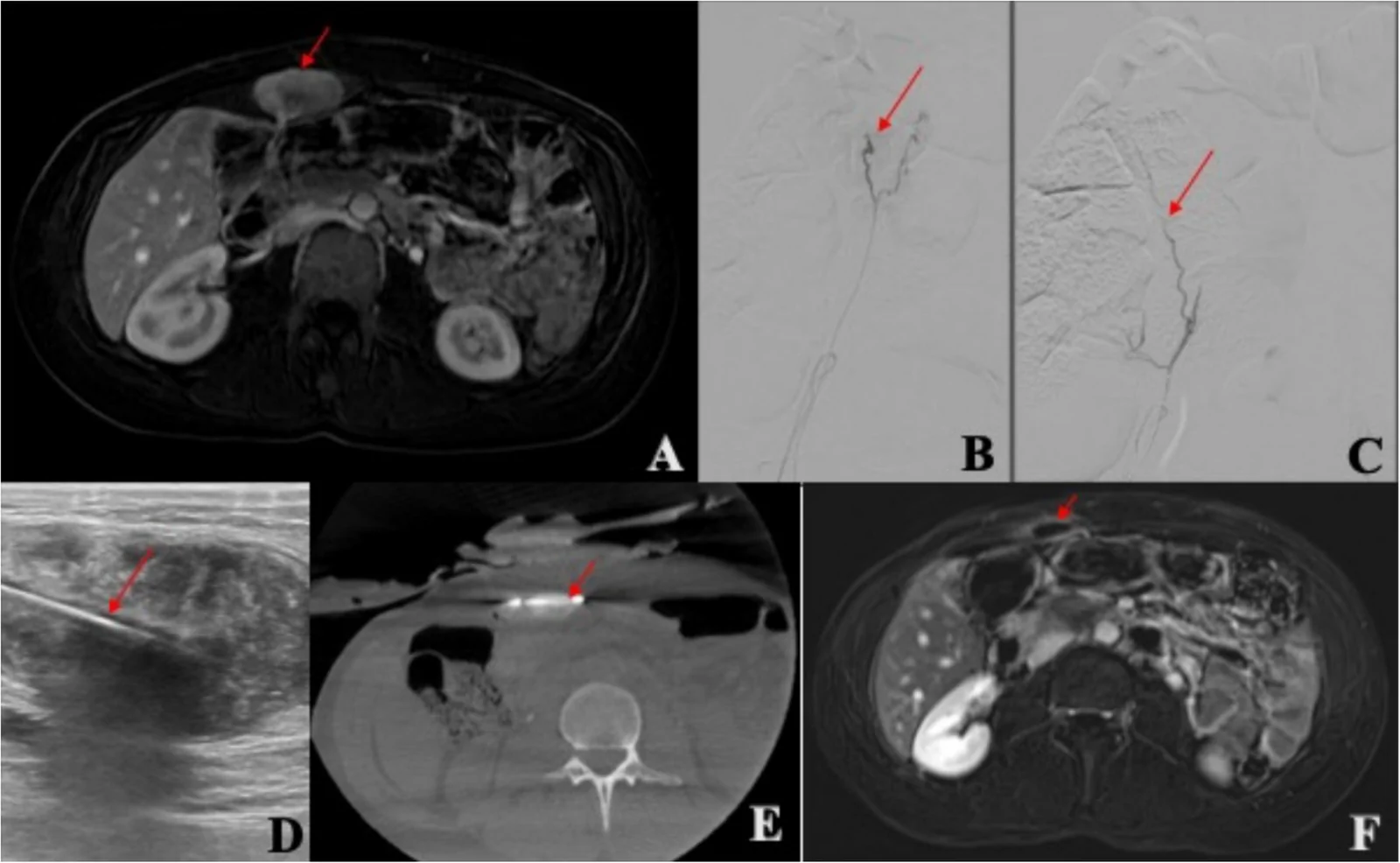
29-year-old woman with an enlarging right abdominal wall desmoid tumor, presenting with baseline dull pain exacerbated by abdominal flexion and interfering with activities of daily living, with pain spiking to 8/10 following activity. The patient underwent staged transarterial embolization and percutaneous cryoablation. **A** Pre-treatment axial contrast-enhanced MRI of the abdomen demonstrating an enhancing right abdominal wall desmoid tumor (arrow). **B** Pre-embolization digital subtraction angiogram showing avid arterial supply from the inferior epigastric artery with a tortuous, dilated feeding vessel (arrow) prior to embolization. **C** Digital subtraction angiogram following embolization with 250 μm Embozene microspheres (Varian Medical Systems, Palo Alto, CA) to stasis, demonstrating successful devascularization of the tumor with stasis of flow within the embolized feeding branch (arrow). **D** Intraprocedural ultrasound image demonstrating ultrasound-guided cryoprobe insertion, with the echogenic probe (arrow) traversing the hypoechoic lesion. **E** Intraprocedural axial CT image during cryoablation demonstrating one of two cryoablation probes (Varian Medical Systems, Palo Alto, CA) in position within the tumor (arrow), with a 10-8-10-4 freeze-thaw-freeze-thaw protocol performed; adjunctive thermoprotective measures including hydrodissection and surface warm-pack application were used to protect adjacent structures. **F** Six-month follow-up axial contrast-enhanced MRI demonstrating resolution of the mass with mild fat stranding adjacent to the prior desmoid bed (arrow), consistent with favorable treatment response. Case courtesy of Junaid Raja, MD

A notable recent development is laparoscopic-assisted percutaneous cryoablation for abdominal wall desmoid tumors, which have historically been difficult to treat because of proximity to intra-abdominal viscera [39] (Fig. 1). Odisho et al. described this approach using pneumoperitoneum-assisted laparoscopic visualization to guide cryoablation of an abdominal wall tumor abutting the colon; insufflation at 15 mmHg created adequate spacing between tumor and bowel, while simultaneous hydrodissection with warm saline and topical warming protected both skin and peritoneal surfaces [40]. This hybrid surgical-interventional approach illustrates how multidisciplinary collaboration can expand treatment eligibility for anatomically challenging locations.

### Irreversible Electroporation

IRE delivers brief, high-voltage electrical pulses to induce cell death via membrane permeabilization while preserving extracellular matrix and neurovascular structures, properties theoretically attractive for desmoid tumors abutting critical structures. The only desmoid-specific data come from Maughan et al., who treated 8 tumors in 6 patients with biopsy-proven aggressive fibromatosis (5 female, 1 male; age range 24–54 years) [41]. All tumors achieved modified Choi partial response, but 5 of 8 tumors eventually met RECIST 1.1 progressive disease criteria at a mean of 335 days. IRE is technically demanding, requiring general anesthesia with neuromuscular blockade and cardiac synchronization. Given the limited evidence and high recurrence rate, IRE remains investigational, best reserved for tumors near critical neurovascular structures where thermal modalities carry prohibitive risk.

### Transarterial Embolization

Although desmoid tumors vary in vascularization [42], hypervascular lesions provide a rationale for transarterial approaches. The NCCN principles of interventional oncology frame catheter-directed therapies as falling into three categories: bland embolization, chemoembolization (conventional and drug-eluting-bead), and radioembolization, each of which warrants consideration in desmoid fibromatosis [13].

### Transarterial Embolization

#### Doxorubicin-Eluting Bead Chemoembolization

Doxorubicin-loaded microspheres delivered into the tumor’s arterial supply combine direct cytotoxic effect with particle-induced ischemia and achieve up to 30-fold higher tissue drug retention with lower plasma concentrations compared with conventional chemoembolization [20, 43]. The NCCN principles specifically endorse DEB-TACE as a reasonable option for selected extra-abdominal desmoid tumors when cryoablation is not technically feasible [13].

In adults, a combined prospective and retrospective study of 24 patients (median tumor size 10.5 cm) demonstrated a median 59% volume decrease at 8 months, although no complete responses were achieved; self-resolving skin ulcers were the most common adverse event [20]. A smaller series of 11 patients reported pain reduction in 90.9%, with complete pain resolution in three [44]. In the pediatric setting, Elnekave et al. treated four children with recurrent or refractory extra-abdominal desmoid tumors using super-selective delivery of doxorubicin-loaded DC Beads, achieving 54–97% volume reduction with cumulative doxorubicin doses as low as 10% of standard intravenous dosing and no cardiotoxicity [43]. Shkalim Zemer et al. combined DEB-TACE with systemic vinblastine and methotrexate in two pediatric patients with large chest wall tumors, with notable tumor shrinkage and neurological improvement [45]. Anatomic applications have extended to unusual locations, including desmoid fibromatosis involving the pubic bone [46]. Tumescent vasoconstriction with dilute epinephrine has been described to minimize cutaneous complications during DEB-TACE of superficial tumors [47].

The pediatric experience is particularly compelling: near-complete responses at substantially lower doxorubicin doses preserve dose capacity for potential future systemic use, an important consideration in a disease characterized by unpredictable recurrence.

### Transarterial Embolization

#### Bland Particle Embolization

Bland embolization using calibrated microspheres, polyvinyl alcohol particles, or gelfoam without a cytotoxic payload relies on ischemic tumor injury alone. Desmoid-specific experience is limited to case reports and small series. It may be particularly appropriate for pediatric patients in whom anthracycline exposure should be minimized or as a cytoreductive adjunct prior to definitive ablation.

### Transarterial Embolization

#### Current Role and Future Directions

DEB-TACE is currently best positioned consistent with emerging literature as an alternative when cryoablation is not technically feasible, as a cytoreductive or palliative approach in adults with large tumors, and as a dose-sparing cytotoxic option in children. Bland and radioembolic approaches remain investigational. Yttrium-90 radioembolization could theoretically deliver localized radiation via the tumor’s arterial supply, but human desmoid experience is absent, and feasibility and safety studies are needed before consideration. Combination strategies such as embolization followed by definitive cryoablation or embolization combined with systemic therapy, represent logical next steps and warrant prospective study (Fig. 1).

### Protection of Adjacent Structures

Key thermoprotective strategies include hydrodissection and pneumodissection, topical warming to prevent cryoablation-related skin injury, strategic multi-probe ice ball sculpting, staged ablation for large or complex tumors, and intraoperative neurophysiologic monitoring for tumors adjacent to critical nerves [15, 48]. These techniques render inability to displace or protect adjacent structures a relative rather than absolute contraindication to ablation [13].

### Beyond Percutaneous Treatment Options

Additional treatment strategies warrant consideration within the global approach to desmoid tumors. Active surveillance is the first-line strategy for primary sporadic disease and remains appropriate in selected cases of progression. For most progressive sporadic tumors, systemic therapy is the next option; chemotherapy has largely been replaced by targeted agents, with nirogacestat (a gamma-secretase inhibitor) and sorafenib (a TKI) now preferred given their high level of evidence and consensus across guidelines.

The role of surgery has evolved substantially. Once the standard treatment, it is no longer routinely recommended because of postoperative recurrence rates of approximately 25–60% even after complete resection [5]. Surgery is now reserved for progressive intra-abdominal or retroperitoneal tumors, as a less-preferred option elsewhere, or for the management of life-threatening complications, with the decision guided by a multidisciplinary team [49].

Percutaneous options should be considered for progressive, symptomatic, morbid, or treatment-refractory extra-abdominal tumors, particularly when local control can improve pain, preserve function, or reduce morbidity while avoiding the adverse effects of surgery or prolonged systemic therapy. This is especially true for localized disease, recurrent tumors after prior therapy, or poor surgical candidates. Careful patient selection is essential, considering the tumor size, proximity to critical neurovascular structures or skin, expected ablation margins, anticipated functional outcomes, and prior treatment [49].

Desmoid tumors are complex lesions with high person-to-person variability in treatment response. Despite efforts to standardize protocols, all cases should be reviewed in a multidisciplinary setting at centers with desmoid expertise, accounting for tumor biology, location, and symptoms [14].

### Interplay with Systemic Therapies

Most patients in the ablation literature have undergone prior systemic therapy before referral for image-guided intervention. The CRYODESMO-01 trial included patients only after failure of two lines of medical therapy [24], and most retrospective cryoablation series enrolled patients previously treated with NSAIDs, hormonal therapy, low-dose chemotherapy, or tyrosine kinase inhibitors [25, 26, 33]. Pediatric patients receiving intra-arterial doxorubicin-eluting embolization had typically failed multiple prior systemic lines [43]. This pattern reflects the traditional positioning of locoregional techniques as salvage therapy, but growing evidence supports earlier use. The NCCN guidelines now recognize ablation both for progression on conventional therapy and as a well-tolerated maintenance strategy after stability on systemic therapy, citing prolonged progression-free and systemic-therapy-free intervals [13]. Pediatric data reinforce this trajectory: 50% of children refractory to methotrexate-vinblastine ultimately require local therapy [50], and local recurrence rates as high as 55% after macroscopically complete resection [51] make percutaneous ablation an attractive alternative to repeat surgery in anatomically complex locations.

### Nirogacestat & Sorafenib

The therapeutic landscape was fundamentally altered by the FDA approval of nirogacestat in November 2023, the first systemic therapy approved for progressing desmoid tumors [52]. In the phase 3 DeFi trial, nirogacestat reduced the risk of progression by 71% versus placebo, with an objective response rate of 41%, and produced significant improvements in patient-reported outcomes [51]. Sorafenib, the principal alternative, demonstrated a two-year progression-free survival of 81% versus 36% for placebo in its phase III trial [53]. Additional agents including AL102, vactosertib, and tegavivint are in development [54].

Both approved agents carry clinically significant toxicities. Nirogacestat is associated with diarrhea (84%), ovarian dysfunction in approximately 75% of women of reproductive potential, rash, and hypophosphatemia [52, 55]. Sorafenib carries risks of rash, hypertension, and hand-foot syndrome [53]. An unresolved but clinically important question is the optimal duration of systemic therapy. Long-term DeFi follow-up (median exposure 33.6 months) demonstrated sustained efficacy with continued nirogacestat treatment, with an objective response rate of 45.7% reported with up to 4 years of treatment; however, the appropriate stopping point remains undefined [56]. Sorafenib similarly requires indefinite dosing in the absence of prospective discontinuation data, although real-world retrospective series suggest that discontinuation after sustained response may be feasible in selected patients [57].

This uncertainty regarding treatment duration directly reinforces the rationale for a “systemic-therapy-sparing” role for percutaneous ablation: if definitive local control can be achieved with cryoablation, patients may avoid the cumulative toxicity and indefinite commitment of systemic therapy, a consideration of particular relevance for young women facing nirogacestat-associated ovarian toxicity.

### Future Concepts: Sequencing and Combination Strategies

**Fig. 2:**
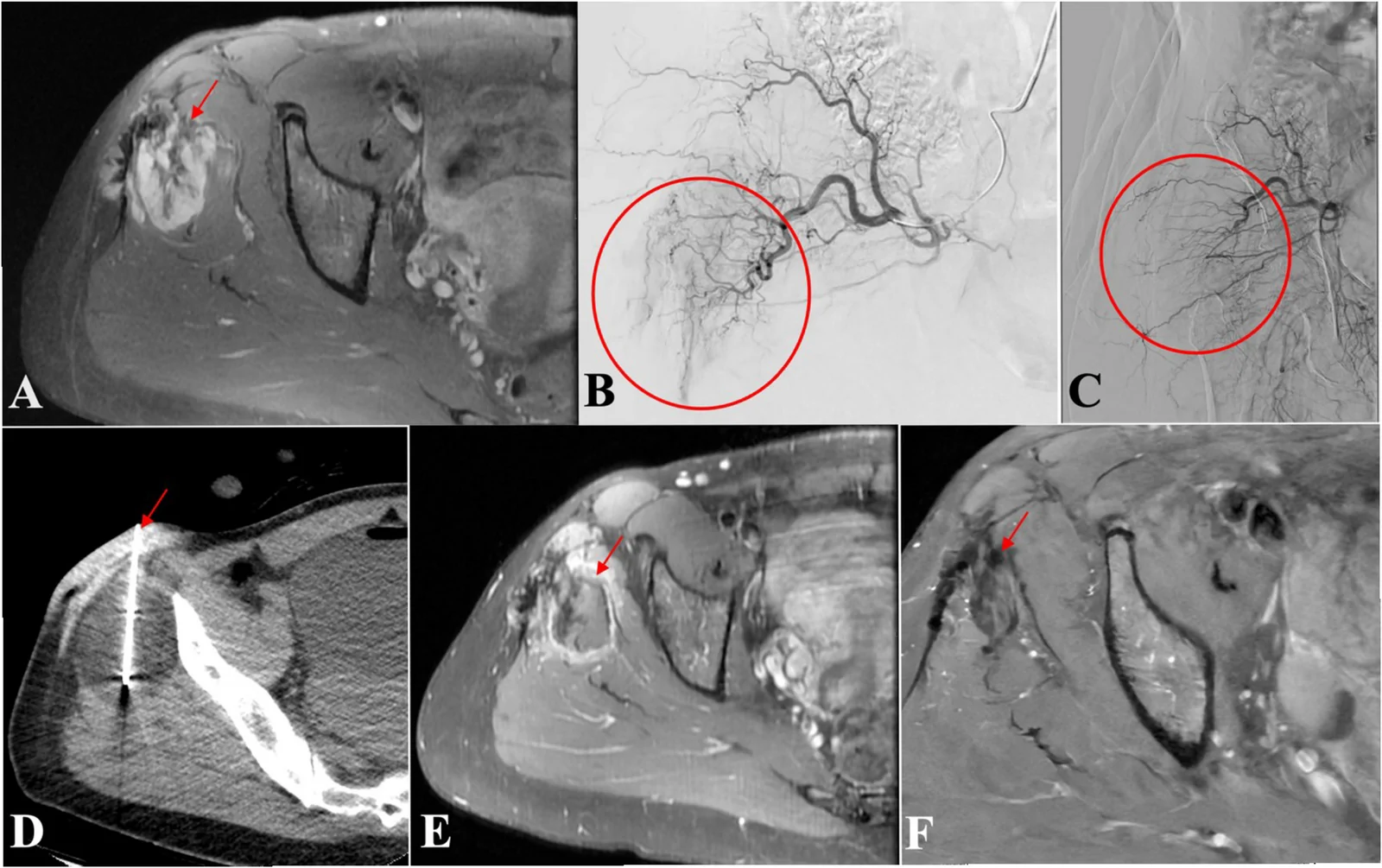
A 52-year-old woman with biopsy-proven desmoid-type fibromatosis of the right anterolateral gluteal region — refractory to four sessions of high-intensity focused ultrasound (HIFU) and sorafenib, with interval growth and right hip pain — underwent staged transarterial embolization and percutaneous cryoablation. **A** Pre-treatment axial T1-weighted post-contrast MRI showing an infiltrative enhancing lesion (arrow) measuring 10.7 × 4.2 × 3.4 cm in the right anterolateral gluteal musculature, primarily involving the proximal gluteus medius and iliotibial band, with mild interval growth from prior imaging. **B** Initial digital subtraction angiogram (DSA) of a selective branch of the right superior gluteal artery demonstrates hypervascular tumor blush consistent with desmoid tumor (circle). Embolization was subsequently performed using 500–700 μm Embosphere microspheres (Merit Medical, South Jordan, UT) until stasis **C** Digital subtraction angiogram (DSA) of the right internal iliac artery obtained after transarterial embolization demonstrates no residual tumor blush (circle) prior to cryoablation. **D** Intraprocedural axial CT image during percutaneous cryoablation, performed in the same session following embolization, demonstrates cryoprobe (arrow) within the lesion, with ice coverage extending beyond the margins of the targeted tumor. **E** Axial T1-weighted post-contrast MRI obtained approximately 3 months after cryoablation, demonstrates interval decrease in size and overall enhancement of the desmoid tumor (arrow), consistent with treatment response. Residual peripheral enhancement is consistent with posttreatment change, with no new nodular or mass-like enhancement. **F** Surveillance axial T1-weighted post-contrast MRI obtained approximately 3.5 years after cryoablation, demonstrating complete response (arrow), with no new suspicious lesions or significant interval growth, consistent with sustained treatment response. Case courtesy of Gina Landinez, MD

The complementary profiles of percutaneous ablation and systemic therapy create logical opportunities for sequencing and combination. The optimal timing of these modalities remains an open question, and ongoing prospective trials should help clarify this. This includes CRYODESMO-02, which permits crossover to cryoablation upon progression on systemic therapy, and a phase II study of nirogacestat combined with cryoablation (NCT05949099). Although the following sequence of treatments are not currently considered standard of care, three clinical scenarios warrant particular consideration based on the limited available evidence and the potential to provide meaningful clinical benefit:

1. Neoadjuvant systemic therapy before cryoablation. In patients with large tumors (> 10 cm) or tumors in anatomically complex locations where complete ice ball coverage is technically challenging, a finite course of nirogacestat or sorafenib may achieve cytoreduction that renders a previously unfavorable tumor suitable for definitive cryoablation. This approach could be particularly valuable for young women of reproductive potential, for whom a time-limited course of nirogacestat followed by definitive local therapy may offer better reproductive outcomes than indefinite systemic treatment.
2. Concurrent systemic therapy with focal ablation. In patients with multifocal or locally extensive disease, concurrent systemic therapy may target subclinical disease while ablation addresses the dominant symptomatic lesion.
3. Ablation as a systemic-therapy-sparing intervention. Consistent with available guidelines concept of ablation as maintenance therapy [13], for patients with oligoprogressive disease on otherwise effective systemic therapy, local cryoablation of the progressing site may permit continued use of the systemic agent rather than premature switching of lines. (Fig. 2)

### Patient Selection and Multidisciplinary Decision-making

A French nationwide survey demonstrated that approximately 29% of desmoid tumors were initially misdiagnosed outside expert centers, and that centralized management improved diagnostic accuracy and guideline adherence [58]. These findings underscore why optimal patient selection for percutaneous ablation requires a multidisciplinary approach involving medical oncology, surgical oncology, interventional radiology, radiation oncology, and pathology [14, 59]. All available guidelines explicitly call for evaluation by a multidisciplinary team with expertise in desmoid tumors before initiating any treatment plan [13, 14].

**Fig. 3:**
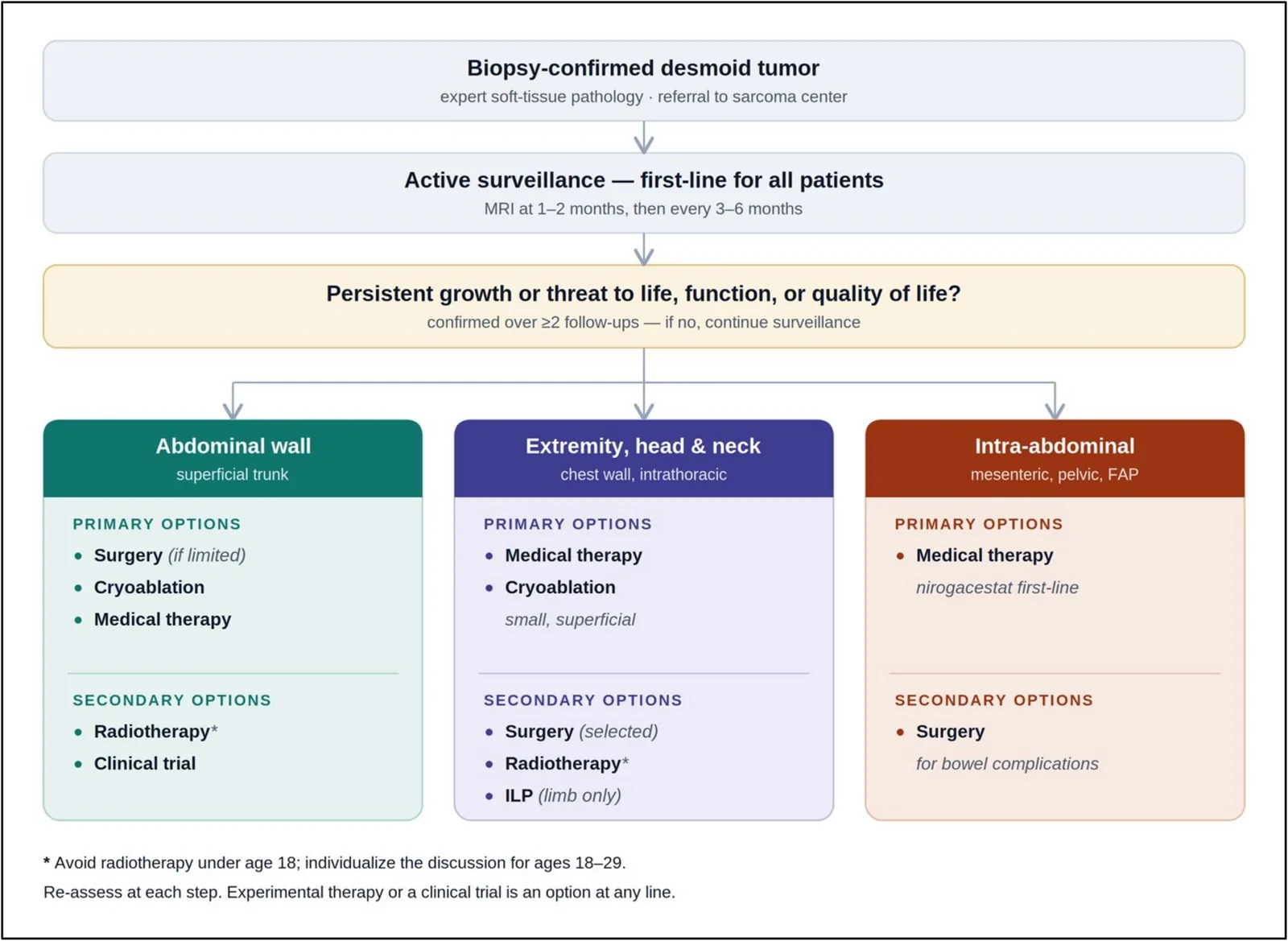
Anatomy- and symptom-based treatment pathway for desmoid tumor, consistent with contemporary multidisciplinary practice. After diagnosis confirmation by an expert soft-tissue pathologist and referral to a sarcoma center, active surveillance with magnetic resonance imaging at 1–2 months and then every 3–6 months is the initial strategy for all patients. Active treatment is initiated only when growth persists over two or more consecutive follow-ups or when the tumor threatens life, function, or quality of life; otherwise, surveillance continues. When treatment is indicated, management branches by anatomic site: abdominal wall and superficial trunk; extra-abdominal soft tissue (extremity, chest wall, head and neck, and intrathoracic); and intra-abdominal disease (mesenteric, pelvic, retroperitoneal, and FAP-associated). Within each branch, primary options are listed in the order of typical consideration, and secondary options are reserved for primary-treatment failure or unfavorable anatomy. Within medical therapy, nirogacestat is preferred first-line when active systemic treatment is indicated, followed by sorafenib; low-dose methotrexate with or without vinorelbine or vinblastine; pazopanib; and liposomal doxorubicin. Because no comparative trials establish a definitive sequence, agent selection integrates each drug’s toxicity profile, age and reproductive considerations, access, and cost. Cryoablation appears among the primary options for accessible extra-abdominal lesions. For adolescent and young adult patients, decisions are highly individualized to weigh the benefits of local and systemic therapy against the distinct risks of late toxicity and secondary malignancy; radiotherapy (asterisk) is avoided in patients younger than 18 years and individualized for those aged 18–29. Failure of a primary local therapy does not preclude further local therapy in the recurrent setting: the decision node is re-entered and anatomy-based selection resumes, with options constrained by the cumulative toxicity and anatomic changes imposed by prior treatment. Experimental therapy or clinical-trial enrollment remains an option at any line. *FAP*, familial adenomatous polyposis; *ILP*, isolated limb perfusion; *MRI*, magnetic resonance imaging

The NCCN algorithm stratifies treatment by anatomic location. For extra-abdominal disease (abdominal wall, pelvis, trunk/extremity, head/neck, or intrathoracic) that is progressive, morbid, or symptomatic, the options include systemic therapy, ablation (with cryoablation as the most utilized modality) or embolization, definitive radiation, and the less-preferred alternative of surgery [13] (Fig. 3). For intra-abdominal or retroperitoneal disease, systemic therapy and (less preferred) surgery are the principal options, reflecting the greater technical difficulty of percutaneous approaches at those sites [13].

Treatment decisions must consider tumor size, location, proximity to critical structures, symptom burden, prior treatment history, and patient preferences. Ablation appears most effective for tumors smaller than 7–10 cm, though larger tumors can be treated with staged procedures or partial ablation for symptom palliation [24, 25]. Because placebo-arm response rates in the sorafenib and nirogacestat trials were 20% and 8% respectively [52, 53], and because spontaneous regression is documented in up to 20% of newly diagnosed desmoid patients [13], patients referred for ablation should ideally have documented radiographic or symptomatic progression to avoid overtreating tumors that may regress without intervention.

Pediatric and adolescent patients warrant particular consideration. Long-term considerations weigh heavily against radiotherapy in this population given the well-documented risk of radiation-associated sarcoma and other late toxicities in growing tissues [60]. Percutaneous ablative techniques, alone or in combination with systemic therapy, may offer durable local control while avoiding the cumulative morbidity associated with radiation exposure over a long-expected survival. The optimal selection of ablative modality, the extent of treatment for large or anatomically complex lesions, and integration with systemic agents remain to be defined, and dedicated pediatric data are limited.

### Comparison with Systemic and Other Local Therapies

With the approval of nirogacestat and the established efficacy of sorafenib, clinicians now face the challenge of selecting and sequencing among multiple effective modalities [52, 53]. Direct comparisons across study designs are not possible, but several distinctions inform decision-making.

Compared with systemic therapy, percutaneous cryoablation achieves broadly comparable local control with the advantage of a single or limited number of sessions rather than indefinite treatment and avoids the systemic toxicity profile of approved agents — most notably the ovarian dysfunction observed in the majority of reproductive-age women receiving nirogacestat [55]. Systemic therapy, however, remains better suited for multifocal or diffuse disease not amenable to focal ablation, and it offers the only realistic option in anatomic locations where percutaneous access is unsafe.

Compared with surgery, percutaneous ablation offers less morbidity, shorter recovery, and the ability to perform repeat treatments, considerations that underpin the NCCN positioning of surgery as the less-preferred option for extra-abdominal desmoid disease amenable to ablation [13]. Peng et al. reported a 5-year recurrence-free survival of only 52.8% in 211 surgically treated patients, with nearly one-third requiring soft tissue reconstruction [5]. Mandel et al. demonstrated comparable disease control between cryoablation and surgery in a propensity-matched analysis without the functional deficits of wide local excision [33].

Compared with radiation therapy, ablation avoids long-term risks of radiation exposure and the potential for radiation-induced sarcoma, an important consideration in a young patient population and one of the principal arguments for cryoablation in pediatric and adolescent disease [60].

### Future Directions

Several areas of active investigation will shape the future role of percutaneous techniques. The randomized CRYODESMO-02 trial will provide pivotal data on cryoablation versus standard medical therapy as first-line treatment and is expected to elevate percutaneous ablation from a guideline-recognized option toward a level of evidence comparable to systemic therapy, potentially reshaping future NCCN recommendations. The SarcAblate phase 1 study (NCT05111964) is exploring HIFU feasibility for soft tissue sarcoma and desmoid tumors in Western centers [50].

Combination strategies represent the next major frontier. Prospective studies of neoadjuvant nirogacestat or sorafenib before cryoablation, concurrent embolization and ablation for large tumors, and ablation as a systemic-therapy-sparing intervention for oligoprogressive disease are all coherent next steps. Each combination addresses a defined clinical scenario where single-modality therapy is suboptimal, very large tumors, highly vascular tumors, and patients constrained by systemic toxicity or reproductive considerations.

### Post Ablation Imaging Surveillance

Post-ablation imaging interpretation is a practical challenge that recent work has begun to address. Cadour et al. demonstrated that low ADC values on diffusion-weighted imaging at one month after cryotherapy represent expected gelatinous necrosis rather than residual tumor, cautioning against misinterpretation as treatment failure [61]. Düx et al. found that the combination of intermediate T2 signal, nodularity, and contrast enhancement on MRI predicted residual disease growth with high specificity (0.94) and accuracy (0.85), offering potential imaging biomarkers to guide surveillance and retreatment decisions [62].

Emerging technologies may further expand the therapeutic arsenal. Histotripsy, a non-invasive, non-thermal modality using focused ultrasound to induce acoustic cavitation and mechanical tissue disintegration has demonstrated feasibility in human liver tumors and animal bone tumor models [63, 64]. Although not yet applied to desmoid fibromatosis, it may offer new options for tumors in locations not well suited to current ablative techniques. IRE and radioembolization similarly warrant formal investigation.

## Limitations

With the exception of CRYODESMO-01 [24, 25], the current literature consists of retrospective single-center series and pooled analysis of heterogenous studies. No randomized trial has compared percutaneous ablation with systemic therapy, surgery, or surveillance, and referral and selection bias likely report improved efficacy. Follow-up durations are often inadequate for a disease characterized by late recurrence.

Technical limitations also warrant emphasis. Durable control depends on complete tumor coverage, which becomes harder as size increases; largest diameter was the only predictor of failure in CRYODESMO-01, and recurrences cluster more commonly at the periphery. Large or anatomically complex lesions may require multiple probes or staged treatment sessions, and tumors near bowel, skin, or neurovascular structures remain challenging despite thermoprotective techniques. Although major complication rates stay below 5%, procedure related morbidity is not negligible, and some patients may need repeat ablation or transition to alternative therapy.

## Conclusions

Percutaneous image-guided ablation is now integral to desmoid tumor management, with prospective and meta-analytic data demonstrating local control comparable to surgery, high rates of symptom relief, and major complication rates below 5%. The inclusion of ablation and embolization in the NCCN Soft Tissue Sarcoma Guidelines Version 3.2026, with cryoablation identified as the most commonly utilized modality and DEB-TACE recognized when cryoablation is not technically feasible [13], represents a significant milestone for the field. Patient selection requires multidisciplinary evaluation weighing tumor characteristics, location, symptom burden, and reproductive considerations. The CRYODESMO-02 trial, together with prospective studies evaluating combination strategies such as embolization followed by cryoablation and nirogacestat combined with cryoablation, will refine optimal sequencing, patient selection, and the potential systemic-therapy-sparing role of local intervention.

## Key References

- Kurtz JE et al., 2021. CRYODESMO-O1: A prospective, open phase II study of cryoablation in desmoid tumour patients progressing after medical treatment. *European Journal of Cancer*.
  - ◦ This multicenter French phase II trial (NCT02476305) of 50 patients with non-abdominopelvic desmoid tumors progressing after at least two lines of medical therapy met its primary endpoint with an 86% non-progression rate at 12 months and median PFS not reached at 31 months of follow-up. Cryoablation was safe (22% grade 3–4 events, no grade 5) and produced durable improvements in Brief Pain Inventory and EQ5D-3L scores, establishing the first prospective evidence for cryoablation as a tissue-sparing local therapy in progressive, heavily pretreated extra-abdominal disease.
- Gounder M et al., 2023. Nirogacestat, a γ-Secretase Inhibitor for Desmoid Tumors. *New England Journal of Medicine*.
  - ◦ The phase III DeFi trial (NCT03785964) randomized 142 adults with progressing desmoid tumors to oral nirogacestat 150 mg twice daily or placebo and demonstrated a 71% reduction in the risk of progression or death (HR 0.29; 95% CI 0.15–0.55; P<0.001), with objective response rates of 41% vs. 8% and significant improvements across all patient-reported outcomes. Despite frequent but mostly low-grade toxicities (diarrhea 84%, ovarian dysfunction in 75% of women of childbearing potential), this trial provided the first high-level randomized evidence supporting a targeted systemic therapy and led to the first FDA-approved treatment for desmoid tumors.
- Huang K et al., 2023. Efficacy and safety of different thermal ablative therapies for desmoid-type fibromatosis: a systematic review and meta-analysis. *Quantitative Imaging in Medicine and Surgery*.
  - ◦ This meta-analysis of 23 studies and 568 patients with 694 desmoid tumors treated by cryoablation, HIFU, or microwave ablation reported pooled rates of 90% symptom relief, 88% local control, 76% non-perfused volume, and only 3% major complications. Although HIFU showed numerically higher local control (99%) than cryoablation (80%) or microwave ablation (78%) (P=0.01), this difference likely reflects selection bias toward superficial tumors with adequate acoustic windows rather than intrinsic modality superiority, as HIFU cohorts systematically exclude deep, bone-adjacent, or gas-shielded lesions that are routinely treated with cryoablation. By quantitatively aggregating fragmented retrospective data across modalities and imaging guidance, this analysis supports the integration of image-guided thermal ablation into multidisciplinary management of progressive or symptomatic desmoid disease and helps reconcile divergent NCCN, ESMO, and Desmoid Tumor Working Group recommendations.

## Data Availability

No datasets were generated or analysed during the current study.
